# Posterior Cortical Atrophy phenotype in a GBA N370S mutation carrier: a case report

**DOI:** 10.1186/s12883-020-02023-5

**Published:** 2021-01-12

**Authors:** Marina Picillo, Sara Scannapieco, Alessandro Iavarone, Monia Ginevrino, Enza Maria Valente, Paolo Barone

**Affiliations:** 1 Center for Neurodegenerative diseases (CEMAND), Department of Medicine, Surgery and Dentistry, Neuroscience section, University of Salerno, Baronissi, Salerno Italy; 2 Neurological and Stroke Unit, Centro Traumatologico Ortopedico Hospital AORN “Ospedali dei Colli”, Naples, Italy; 3 Università Cattolica del Sacro Cuore, Fondazione Policlinico Universitario “A. Gemelli” IRCCS, Roma, Italy; 4 Laboratory of Medical Genetics, Bambino Gesù Children’s Hospital, Rome, Italy; 5 Department of Molecular Medicine, University of Pavia, Pavia, Italy; 6 IRCCS Mondino Foundation, Pavia, Italy

**Keywords:** Genetics, Parkinsonism, GBA, Posterior cortical atrophy, Case report

## Abstract

**Background:**

Glucocerebrosidase (*GBA*) heterozygous variants are the most important genetic risk factor for the development of alpha-synucleinopathies (i.e., Parkinson’s disease and Dementia with Lewy Bodies). Herein, we report for the first time on a patient with a clinical diagnosis of Posterior Cortical Atrophy, carrier of the common *GBA* heterozygous variant N370S (c.1226A > G).

**Case presentation:**

A 44-year-old woman with positive familial history for Dementia with Lewy Bodies disclosed three related signs characterizing the Balint’s syndrome: ocular apraxia, optic ataxia and simultanagnosia. Over 2-year follow up, overt gaze apraxia (psychic paralysis of gaze) appeared leading to functional blindness. Given her young age at onset and positive familial history, she underwent a next-generation-sequencing (NGS) based screening of a panel of 32 genes related to neurodegenerative conditions within the ANAMNESYS (An origiNal Approach to study faMiliarity in NEurodegenerative SYndromeS) study. NGS demonstrated the N370S variant in the *GBA* gene (rs76763715), confirmed by Sanger sequencing. This is a relatively common variant, with predicted mild impact, already reported to occur in 2.4% of PD Italian patients; however, neither this nor other *GBA* variants have ever been reported to date in patients with Posterior Cortical Atrophy. Glucocerebrosidase activity was investigated and found to be significantly reduced (4.72 nmol/h/mg) compared to healthy controls as well as patients affected by neurodegenerative diseases, further supporting pathogenicity of the *GBA* variant.

**Conclusions:**

We report on a patient with a clinical diagnosis of Posterior Cortical Atrophy, carrier of the *GBA* heterozygous variant N370S (c.1226A > G; p.Asn409Ser) determining reduced GCase activity. This report also confirms the role of NGS-based targeted gene analysis in detecting peculiar clinical phenotypes associated with known pathogenic mutations and reinforces the knowledge that carriers of genetic variants often present phenotypic overlaps across different neurodegenerative syndromes, highlighting the limitations of current clinical diagnostic criteria in defining boundaries between distinct conditions and the difficulties of clinicians in reaching the best clinical diagnosis.

**Supplementary Information:**

The online version contains supplementary material available at 10.1186/s12883-020-02023-5.

## Background

Glucocerebrosidase (*GBA*) heterozygous variants are the most important genetic risk factor for the development of Parkinson’s disease (PD) and Dementia with Lewy Bodies (DLB) (i.e., alpha-synucleinopathies) [1]. Yet, other clinical phenotypes have been associated with *GBA* mutations including Progressive Supranuclear Palsy (PSP) [2, 3].

Herein, we report on a patient with Posterior Cortical Atrophy (PCA), carrier of the common *GBA* heterozygous variant N370S (c.1226A > G).

## Case presentation

**Fig. 1:**
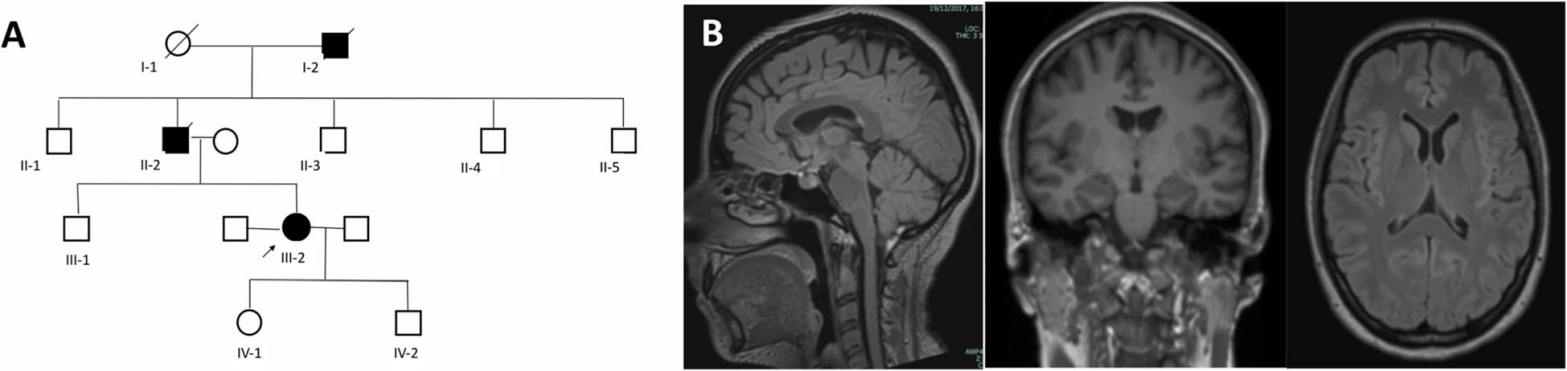
**a**: Pedigree showing autosomal dominant inheritance. I-2: deceased in his 30s and affected by behavioural disturbances. The proband reports paranoid behaviour in the grandfather preceding a bedridden state with death occurring in few years. No medical documentation available. II-2: deceased in his 70s. Affected by Dementia with Lewy bodies diagnosed by a neurologist since the age of 60. Medical records documented a progressive dementia accompanied by fluctuation in cognition, hallucination and spontaneous parkinsonism. Agitation as well as visual hallucinations were worsened by dopaminergic therapy (levodopa) and required treatment with antipsychotic (clozapine).; III-2: the proband. **b**: Proband’s brain MRI showing unremarkable findings


A 44-year-old woman with a 3-year history of vivid dreams presented progressive deterioration of visuo-spatial and visuo-motor abilities despite good visual acuity. No visual hallucinations were reported. Family history was positive for parkinsonism and cognitive impairment (fig. 1a). Mental examination revealed a Mini-Mental State Examination score of 15 with agraphia and impairment in visuo-spatial functions (detailed cognitive evaluation is shown in supplemental material). The patient disclosed three related signs characterizing the Balint’s syndrome: ocular apraxia (i.e., the neuropsychologic inability to shift attention by looking away from an object to one located in the periphery of vision), optic ataxia (i.e., the inability to use visual information to guide the hands accurately for reaching and other activities) and simultanagnosia (i.e., the inability to attend simultaneously to multiple objects in the field of vision). She also showed increased muscle tone with mild left bradykinesia (Video 1).

**Additional file 1 Video 1**. Mild left bradykinesia. Slow saccades with no evident gaze limitation.

Brain MRI was unremarkable (fig. 1b). ^18^F-FDG PET showed bilateral occipito-temporo-parietal hypometabolism with left predominance (images not available), while ^18^F-Florbetapir PET did not demonstrate amyloid deposits. SPECT DAT Scan showed lack of nigro-striatal involvement [right striatum uptake: 2.43; left striatum uptake: 2.47, values expressed in striatal binding ratio (SBR); based on a database of age-matched healthy subjects from the Parkinson Progression Markers Initiative, SBR z score ≥ − 2 are classified as without dopaminergic deficit (ppmi-info.org/data)]. Both PET studies were performed after 3 years of disease history, while SPECT DAT Scan was performed after 6 years of disease history. Despite several trials with different medications including levodopa, rivastigmine, memantine her clinical condition worsened. Over 2-year follow up, overt gaze apraxia (psychic paralysis of gaze) appeared leading to functional blindness, while increased tone and mild left bradykinesia remained stable. She never developed overt visual hallucinations, cognitive or motor fluctuations or psychosis. However, she continued to report vivid dreams and few episodes of persisting vivid dreams during awakening possibly suggesting sleep onset of REM episodes (although a formal sleep study has not been performed). The cognitive profile showing constructional apraxia with difficulties with clock and pentagons would be consistent with DLB. Furthermore, the Benton test was normal indicating unimpaired visuoperceptual function which is usually affected in classical early PCA. From the neuropsychological perspective, we would interpret this apparent discrepancy in terms of the different cognitive functions involved in performing a relatively “pure” visuo-perceptual task (i.e., the Benton’s test), in contrast with tests requiring a complex visuo-motor integration. From the clinical standpoint, patients with “focal” Balint syndrome exhibit non-homogeneous pattern of impairment, in which the three main signs of the triad are not represented to the same extent. This is also true when the syndrome relies on neurodegenerative mechanisms. In such cases some signs and symptoms may be prominent or relatively isolated at onset, before showing the progressive association with other signs able to complete the full picture of the syndrome. However, after excluding a diagnosis of DLB and based on the clinical findings the patient was diagnosed with PCA [4, 5].

The patient underwent a next-generation-sequencing (NGS) based screening of a panel of 32 genes related to neurodegenerative conditions within the ANAMNESYS (An origiNal Approach to study faMiliarity in NEurodegenerative SYndromeS) study (Additional file 1). Genomic DNA was used to prepare an amplicon-based targeted library with the HaloPlex PCR Target Enrichment System (Agilent Technologies), which was run on a MiSeq instrument (Illumina). Data were subjected to bioinformatic analysis and filtering in order to exclude common variants (frequency > 1% in gnomAD database) as well as synonymous and noncoding variants not predicted to impact on splicing. NGS demonstrated the N370S variant in the *GBA* gene (rs76763715), confirmed by Sanger sequencing. This is a relatively common variant, with predicted mild impact, already reported to occur in 2.4% of PD Italian patients; however, neither this nor other *GBA* variants have ever been reported to date in patients with PCA [1]. The same variant was not detected in the eldest unaffected sibling (III-I). Glucocerebrosidase (GCase) activity was investigated in the proband as reported, and was found to be significantly reduced (4.72 nmol/h/mg) compared to healthy controls as well as patients affected by neurodegenerative diseases (range in healthy controls: 7.7–17.47 nmol/h/mg) [6–8].

## Discussion and conclusions

We report on a patient with a clinical diagnosis of PCA, carrier of the *GBA* heterozygous variant N370S (c.1226A > G; p.Asn409Ser) determining reduced GCase activity.

PCA includes a wide and heterogeneous spectrum of neurodegenerative syndromes characterized by visuo-spatial and visuo-perceptual impairment, alexia as well as Balint’s and Gerstmann’s syndrome with gradual progression to dementia and underlined by different pathology [i.e., Alzheimer’s Disease (AD), Corticobasal degeneration or Lewy bodies] [4]. To the best of our knowledge, several observations support the clinical diagnosis of PCA and exclude other neurodegenerative dementias. First, the patient presents the classical Balint’s triad with slow progression over time and fulfils clinical criteria for PCA diagnosis [4]. Then, AD was ruled out by the lack of amyloid deposits shown by ^18^F-Fluorbetapir PET. Similarly, DLB was excluded for the lack of essential core features (i.e., fluctuating cognition and visual hallucinations). As a matter of fact, mild features suggesting a spontaneous parkinsonism were detected, although SPECT DAT Scan revealed integrity of nigro-striatal circuits.

Although our observation lacks of pathological confirmation and, thus, remains speculative, we can hypothesize that, in our patient, PCA is underlined by either Lewy body disease or corticobasal degeneration or mixed pathology. As a matter of fact, positive familial history would suggest a genetic variant with autosomal inheritance pattern as underlying cause of the proband’s neurological picture. Unfortunately, no other unaffected alive siblings consented to genetic testing. It is also possible that nigro-striatal involvement is not evident yet, given the peculiar phenotype of disease. Despite being relatively common in healthy population (0.36% frequency in Italy) [9], the pathogenic impact of N370S is well established and indeed GCase enzymatic is significantly reduced in our patient as well as in other mutation carriers [6–8]. Although we did not perform whole exome sequencing, yet we excluded point mutations in the most relevant candidate genes for neurodegenerative disorders (including *SNCA*) as well as CNV in *SNCA* gene with MLPA analysis and it is very unlikely that a broader genetic test such as exome sequencing would have revealed variants of clear pathogenic significance in other genes. Additionally, we are not aware of other in vivo biomarkers possibly supporting a better clinical classification of our patient.

This report also confirms the role of NGS-based targeted gene analysis in detecting peculiar clinical phenotypes associated with known pathogenic mutations and reinforces the knowledge that carriers of genetic variants often present phenotypic overlaps across different neurodegenerative syndromes, highlighting the limitations of current clinical diagnostic criteria in defining boundaries between distinct conditions and the difficulties of clinicians in reaching the best clinical diagnosis.

## Supplementary Information

**Additional file 2.** List of the genes included in the NGS panel and detailed cognitive evaluation.

## Data Availability

Data sharing is not applicable to this article as no datasets were generated or analysed during the current study.

## Abbreviations

AD: Alzheimer’s disease
DLB: Dementia with Lewy bodies
GBA: Glucocerebrosidase
MRI: Magnetic resonance imaging
NGS: Next generation sequencing
PCA: Posterior cortical atrophy
PD: Parkinson’s disease
PET: Positron emission tomography
PSP: Progressive Supranuclear Palsy
SPECT: Single photon emission computerized tomography
