# Supplementary material for: Posterior Cortical Atrophy phenotype in a GBA N370S mutation carrier: a case report
Source: BMC Neurol. 2021 Jan 12;21:17. doi: 10.1186/s12883-020-02023-5 (PMC7802182; doi:10.1186/s12883-020-02023-5)
Supplement: Supplementary file 2 — Additional file 2. List of the genes included in the NGS panel and detailed cognitive evaluation. [file 12883_2020_2023_MOESM2_ESM.docx]

Genes included in the NGS panel

| **Number** | **Gene name** | **Genomic coordinates (GRCh37)** | **Cytogenetic location** | **OMIM** |
| --- | --- | --- | --- | --- |
| **1** | ADORA1 | Chr1:203,059,782-203,136,533 | 1q32.1 | 102775 |
| **2** | APP | Chr21:27,252,861-27,543,446 | 21q21.3 | 104760 |
| **3** | ATP13A2 | Chr1:17,312,453-17,338,423 | 1p36.13 | 610513 |
| **4** | ATP1A3 | Chr19:42,470,734-42,501,649 | 19q13.2 | 182350 |
| **5** | CHMP2B | Chr3:87,276,421-87,304,698 | 3p11.2 | 609512 |
| **6** | DCTN1 | Chr2:74,588,281-74,619,214 | 2p13.1 | 601143 |
| **7** | DNAJC6 | Chr1:65,713,902-65,881,552 | 1p31.3 | 608375 |
| **8** | FBXO7 | Chr22:32,870,663-32,894,818 | 22q12.3 | 605648 |
| **9** | FUS | Chr16:31,191,431-31,203,127 | 16p11.2 | 137070 |
| **10** | GBA | Chr1:155,204,243-155,214,490 | 1q22 | 606463 |
| **11** | GCH1 | Chr14: 55,308,726-55,369,570 | 14q22.2 | 600225 |
| **12** | GRN | Chr17:42,422,614-42,430,470 | 17q21.31 | 138945 |
| **13** | HNRNPA2B1 | Chr7:26,229,547-26,241,149 | 7p15.2 | 600124 |
| **14** | MAPT | Chr17:43,971,748-44,105,700 | 17q21.31 | 157140 |
| **15** | NPC1 | Chr18:21,086,148-21,166,862 | 18q11.2 | 607623 |
| **16** | NPC2 | Chr14:74,942,895-74,960,880 | 14q24.3 | 601015 |
| **17** | PARK2 | Chr6:161,768,452-163,148,803 | 6q26 | 602544 |
| **18** | PARK7 | Chr1:8,014,351-8,045,565 | 1p36.23 | 602533 |
| **19** | PINK1 | Chr1:20,959,948-20,978,004 | 1p36.12 | 608309 |
| **20** | PLA2G6 | Chr22:38,507,502-38,601,697 | 22q13.1 | 603604 |
| **21** | PODXL | Chr7:131,185,021-131,242,976 | 7q32.3 | 602632 |
| **22** | PRKRA | Chr2:179,296,141-179,316,239 | 2q31.2 | 603424 |
| **23** | PSEN1 | Chr14:73,603,126-73,690,399 | 14q24.2 | 104311 |
| **24** | PSEN2 | Chr1:227,057,885-227,083,806 | 1q42.13 | 600759 |
| **25** | RAB39B | ChrX:154,487,526-154,493,874 | Xq28 | 300774 |
| **26** | SMPD1 | Chr11:6,411,655-6,416,228 | 11p15.4 | 607608 |
| **27** | SNCA | Chr4:90,645,250-90,759,466 | 4q22.1 | 163890 |
| **28** | SQSTM1 | Chr5:179,233,388-179,265,078 | 5q35.3 | 601530 |
| **29** | SYNJ1 | Chr21:34,001,069-34,100,359 | 21q22.11 | 604297 |
| **30** | TARDBP | Chr1:11,072,414-11,085,796 | 1p36.22 | 605078 |
| **31** | VCP | Chr9:35,056,061-35,073,246 | 9p13.3 | 601023 |
| **32** | VPS13C | Chr15:62,144,588-62,352,672 | 15q22.2 | 608879 |

First cognitive evaluation after 3-year disease duration

|  | **Raw score** | **Corrected score** | **Equivalent score** | **Outcome** | **Explored cognitive function** |
| --- | --- | --- | --- | --- | --- |
| Mini Mental State Examination (cut-off 23,80) | 15 | 14.62 |  | **Pathological** | ***Global cognition*** |
| Raven’s matrices | 10 | 9.3 | 0 | **Pathological** | ***Analogical reasoning, capacity for abstraction*** |
| The Rey 15 Item Test  – immediate recall | 9 | 5.5 | 0 | **Pathological** | ***Short- and long-term verbal memory*** |
| The Rey 15 Item Test  – delayed recall | 0 | 0.20 | 0 | **Pathological** |  |
| The Rey 15 Item Test - recognition | 1/15 |  |  |  |  |
| 15 parole di Rey – false recognition | 0/31 |  |  |  |  |
| Prose memory | 11.1 | 10.1 | 2 | Within normal range |  |
| Digit Span counterclock | 3 | 2.90 | 1 | *Inferior limit of normal range* | ***Working memory*** |
| Digit Span | 4 | 3.82 | 0 | **Pathological** | ***Short-term memory*** |
| Verbal Span | 2 | 1.50 | 0 | **Pathological** |  |
| Corsi | 2 | 1.75 | 0 | **Pathological** |  |
| Rey’s Figure - delayed recall | 9 | 8.25 | 0 | **Pathological** | ***Spatial long-term memory*** |
| Rey’s Figure - Copy | 18 | 17.25 | 0 | **Pathological** | ***Executive functions*** |
| Clock drawing test - total | 7 | 6.77 | 1 | *Inferior limit of normal range* |  |
| Clock drawing test - numbers | 3 | 2.89 | 2 | Within normal range |  |
| Clock drawing test - hands | 2 | 1.90 | 1 | *Inferior limit of normal range* |  |
| Phonemic fluency | 15 | 15.7 | 0 | **Pathological** |  |
| Semantic fluency | 20 | 19 | 0 | **Pathological** |  |
| Trail Making Test: A | 183 | 179 | 0 | **Pathological** |  |
| Trail Making Test: B | 730 | 713 | 0 | **Pathological** |  |
| Trail Making Test: B-A | 547 | 533 | 0 | **Pathological** |  |
| FAB | 7 | 6.7 | 0 | **Pathological** |  |
| Stroop Test (inhibitory control) | 6 | 3.30 | 0 | **Pathological** |  |
| Stroop Test (reading) | 34 |  |  |  | ***Language*** |
| Stroop Test (denomination) | 19 | 15.30 | 0 | **Pathological** |  |
| Denomination of nouns (ENPA) | 10 | 10 |  | Within normal range |  |
| Denomination of verbs (ENPA) | 9 | 8.9 |  | Within normal range |  |
| Constructional apraxia (Milano) | 5 | 4 | 0 | **Pathological** | ***Visuo-spatial an praxyc abilities*** |
| Benton test | 18 | 20 |  | Within normal range | ***Visuo-spatial perception*** |
| ADL | 5/6 |  |  |  | ***Activities of daily living*** |
| IADL | 5/8 |  |  |  | ***Instrumental sctivities of daily living*** |
